# Antimicrobial Peptides from Fish

**DOI:** 10.3390/ph7030265

**Published:** 2014-03-03

**Authors:** Jorge A. Masso-Silva, Gill Diamond

**Affiliations:** 1 Department of Pediatrics and Graduate School of Biomedical Sciences, Rutgers New Jersey Medical School, Newark, NJ 07101, USA; E-Mail: massoja@gsbs.rutgers.edu; 2 Department of Oral Biology, University of Florida, Box 100424, Gainesville, FL 32610, USA

**Keywords:** defensin, pleurocidin, cathelicidin, hepcidin, piscidin

## Abstract

Antimicrobial peptides (AMPs) are found widely distributed through Nature, and participate in the innate host defense of each species. Fish are a great source of these peptides, as they express all of the major classes of AMPs, including defensins, cathelicidins, hepcidins, histone-derived peptides, and a fish-specific class of the cecropin family, called piscidins. As with other species, the fish peptides exhibit broad-spectrum antimicrobial activity, killing both fish and human pathogens. They are also immunomodulatory, and their genes are highly responsive to microbes and innate immuno-stimulatory molecules. Recent research has demonstrated that some of the unique properties of fish peptides, including their ability to act even in very high salt concentrations, make them good potential targets for development as therapeutic antimicrobials. Further, the stimulation of their gene expression by exogenous factors could be useful in preventing pathogenic microbes in aquaculture.

## 1. Introduction

Antimicrobial peptides (AMPs) represent a broad category of different families of highly conserved peptides widely found throughout Nature, which exhibit broad-spectrum antimicrobial activity *in vitro* and *in vivo*. While vertebrate antimicrobial peptides were initially discovered in amphibians, humans and rabbits in the mid-1980s [1,2,3], The antimicrobial activity of fish peptides was not described for another decade. Initially, a toxic peptide from the Moses sole fish *Pardachirus marmoratus*, called pardaxin, was characterized in 1980 [4], but its antimicrobial activity wasn’t observed until 1996 [5]. Shortly thereafter, Cole *et al*. described a peptide isolated from the skin secretions of the winter flounder (*Pleuronectes americanus*) [6] using antimicrobial activity as a screening method. Since then, the field has progressed as with other vertebrate species, with the identification of homologous peptides in the piscidin family (unique to fish, but homologous to cecropins), as well as the defensin, cathelicidin, and hepcidin families, which are found in many other species. Many of the peptides were identified by purification of the peptides with antibiotic activity, although as with other species, the increased use of bioinformatics techniques has allowed the identification of even more peptides [7]. The results of the research described here demonstrate that AMPs from fish exhibit many if not all of the same characteristics as other vertebrate AMPs, like broad-spectrum (but often species-specific) antimicrobial activities, as well as immunomodulatory functions. In addition, there appear to be interesting differences, specific to fish, that have evolved to address the unique aquatic environments and microbes encountered by these species. There has also been a recent effort to study the potential for using these peptides as therapeutic agents, both in human medicine and in aquaculture. Below we will examine the various peptide families (whose members are shown in Table 1), and discuss their role in host defense and potential for future use.

**Table 1:** Characterized antimicrobial peptides from fish, by species. Listed is the number of peptides of each family [reference].

Species			Piscidins	Defensins	Hepcidins	Cathelicidins	Histone-derived
Common name	Scientific name	Habitat					
American plaice	*Hippoglossoides platessoides*	Marine	2 [8]				
Antarctic toothfish	*Dissostichus mawsoni*	Marine			3 [9]		
Atlantic cod	*Gadus morhua*	Marine and brackish	2 [10]	1 [11]	1 [12]	1 [13]	1 [14]
Antarctic eelpout	*Lycodichthys dearborni*	Marine			2 [9]		
Atlantic hagfish	*Myxine glutinosa*	Marine				3 [15]	
Atlantic salmon	*Salmo salar*	Marine, brackish and freshwater			2 [16]	2 [17]	1 [18]
Ayu	*Plecoglossus altivelis*	Marine, brackish and freshwater			1 [19]	1 [20]	
Barramundi	*Lates calcarifer*	Marine, brackish and freshwater			2 [21]		
Black porgy	*Acanthopagrus schlegelii*	Marine and brackish			7 [22,23]		
Black rockfish	*Sebastes schlegelii*	Marine			2 [24]		
Blotched snakehead	*Channa maculata*	Freshwater			1 [25]		
Blue catfish	*Ictalurus furcatus*	Freshwater and brackish			1 [26]		
Blunt snout bream	*Megalobrama amblycephala*	Freshwater			1 [27]		
Brook trout	*Salvelinus fontinalis*	Marine, brackish and freshwater				2 [28]	
Brown trout	*Salmo trutta fario*	Marine, brackish and freshwater				1 [28]	
Channel catfish	*Ictalurus punctatus*	Freshwater			1 [26]		1 [29]
Chinese loach	*Paramisgurnus dabryanus*	Freshwater		1 [30]			
Common carp	*Cyprinus carpio* L.	Freshwater and brackish		2 [31]	1 [32]		
European seabass	*Dicentrarchus labrax*	Marine, brackish and freshwater	1 [33]		1 [34]		
Gilthead seabream	*Sparus aurata*	Marine and brackish		1 [35]	1 [36]		
Grayling	*Thymallus thymallus*	Freshwater and brackish				1 [28]	
Half-smooth tongue sole	*Cynoglossus semilaevis*	Marine, brackish and freshwater			1 [37]		
Atlantic halibut	*Hippoglossus hippoglossus*	Marine	1 [8]				1 [38]
Hybrid striped bass	*Morone saxatilis x M. chrysops*	Marine, brackish and freshwater	4 [39,40,41]		1 [42]		
Icefish	*Chionodraco hamatus*	Marine	1 [43]				
Olive flounder	*Paralichthys olivaceus*	Marine			2 [16]		
Japanese rice fish	*Oryzias latipes*	Freshwater and brackish			1 [16]		
Japanese pufferfish	*Takifugu rubripes*	Marine, brackish and freshwater		1 [44]			
Japanese seabass	*Lateolabrax japonicus*	Marine, brackish and freshwater			1 [45]		
Largemouth bass	*Micropterus salmoides*	Freshwater			2 [46]		
Large yellow croaker	*Pseudosciaena crocea*	Marine and brackish	1 [47]		1 [48,49]		
Mandarin fish	*Siniperca chuatsi*	Freshwater	1 [50]	1 [51]			
Maori chief	*Notothenia angustata*	Marine			5 [9]		
Medaka	*Oryzias melastigma*	Freshwater and brackish		1 [52]	2 [53]		
Miiuy croaker	*Miichthys miiuy*	Marine and brackish			1 [54]		
Mud dab	*Limanda limanda*	Marine	1 [55]				
Mud loach	*Misgurnus mizolepis*	Freshwater			[56]		
Olive flounder	*Paralichthys olivaceus*	Marine		5 [57]			
Orange-spotted grouper	*Epinephelus coioides*	Marine and brackish	1 [58]	2 [59,60]	3 [61,62]		
Pacific mutton hamlet	*Alphestes immaculatus*	Marine			1 [63]		
Rainbow trout	*Oncorhynchus mykiss*	Marine, brackish and freshwater		4 [64,65]		2 [17]	3 [66,67,68,69]
Redbanded seabream	*Pagrus auriga*	Marine			4 [70]		
Red sea bream	*Chrysophrys major*	Marine	1 [71]		1 [72]		
Rockbream	*Oplegnathus fasciatus*	Marine			4 [73]		
Sea bass	*Dicentrarchus labrax*	Marine, brackish and freshwater					1 [74]
Seahorse	*Hippocampus kuda*	Marine and brackish	1 [75]				
Smallmouth bass	*Micropterus dolomieu*	Freshwater			2 [46]		
Snowtrout	*Schizothorax richardsonii*	Freshwater			1 [76]		
Spotted-green pufferfish	*Tetraodon nigroviridis*	Freshwater and brackish		2 [44]			
Sunshine bass	Marine, brackish and freshwater						1 [69]
Thick-lipped lenok	*Brachymystax lenok*	Freshwater				1 [77]	
Tilapia	Oreochromis mossambicus	Freshwater and brackish	5 [78]		3 [79]		
Turbot	*Scophthalmus maximus*	Marine and brackish			2 [80,81]		
Winter flounder	*Pleuronectes* *americanus*	Marine	6 [6,82,83]		5 [16]		
Witch flounder	*Glyptocephalus cynoglossus*	Marine	5 [8]				
Yellowtail flounder	*Pleuronectes ferruginea*	Marine	1 [8]				
Zebrafish	*Danio rerio*	Freshwater		3 [44]	2 [84]		

## 2. Piscidins

**Figure 1:**
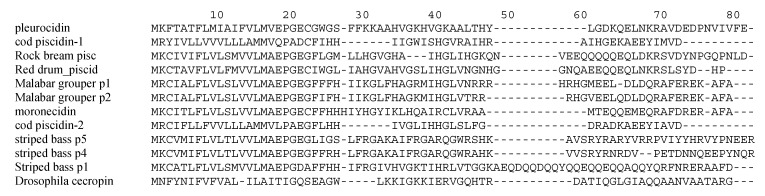
Alignment of piscidins. Mature peptide sequences were obtained from published data and from the PubMed protein database, and were aligned using MacVector software. The Drosophila cecropin A1 sequences is provided for comparison as a representative member of the cecropin family.

Piscidins and pleurocidins comprise a family of linear, amphipathic AMPs, evolutionarily related to similarly structured peptides found in amphibian skin and insects [85]. The first member of the family identified was a 25-residue peptide isolated and characterized from skin mucous secretions of the winter flounder, *Pleuronectes americanus*, called pleurocidin [6]. Further research identified other homologous pleurocidins in related species [8,83]. These were shown to exhibit an amphipathic, α-helical structure, similar to magainins and cecropins. A similarly structured peptide was identified in the loach, *Misgurunus anguillicaudatus*, called misgurin [86], and a family of peptides, termed piscidins, were identified in the mast cells of the hybrid striped bass [87], as well as numerous other fish taxa [88]. Other similar peptides, including moronecidin, epinecidin, dicentracin, have been identified [33,41,89]. An alignment of primary amino acid sequences of some members of this class are shown in Figure 1. The similarities of the mature peptide predicted secondary structure [6,41,78,90] as well as an analysis of their gene structures [33,50] suggested that they all belong to the same evolutionarily related family, which we will refer to as the piscidins. In addition, positive selection has been found influencing evolution of these peptides, where the highest diversity is found in the mature peptide that suggest adaptation for attacking new pathogens or strains that are coevolving with the host [91,92].

Alignment of primary amino acid sequences shows that piscidins as a group have little direct sequence identity (Figure 1), but are as a group predicted to possess an amphipatic α-helical structure [6,41,78,90]. However, CD spectroscopy of the piscidin from brooding pouch suggests that it might have a β-sheet or β-strand motif instead of α-helix [75]. Their gene structure is composed of four exons and three introns, encoding a peptide precursor containing a signal peptide, a mature piscidin and a carboxy-terminal prodomain [41,50,82]. However, in tilapia and grouper a three-exon/two-intron and five-exon/four-intron structure, respectively was found [78,89]. Moreover, multiple piscidin isoforms have been found in the same species [78,83].

Piscidins exhibit potent antimicrobial activity against a variety of microorganisms. They are widely active against bacteria Gram-positive and -negative species, with the best antibacterial values obtained against several *Streptococcus*, *Pseudomonas*, *Bacillus* and *Vibrio* species (for a full listing of fish antimicrobial peptides and their activities, see Supplementary Table S1). Interestingly, chrysophsin-3 was observed to kill the three stages of *Bacillus anthracis* (sporulated, germinated and vegetative), being able to penetrate and kill the spores without full germination [93]. Piscidins have also been shown to possess anti-fungal activity [47,94,95], anti-parasitic activity [47,96,97,98], and anti-viral activity [99,100]. An interesting study showed that piscidin-2 was highly potent against the water mold *Saprolegnia* sp. (Oomycetes) with a MIC within the physiological piscidin-2 levels [98].

Piscidins are mainly expressed in gill, skin and intestine, although can be also found in head-kidney and spleen [10,43,47,50,58,101,102]. However, in Atlantic cod piscidin was found to be ubiquitous, being detected in chondrocytes, heart, oocytes, exocrine and endocrine glands, swim bladder, and other tissues [103]. Nevertheless, the expression profiles vary depending on the isoform [39,78,83]. Moreover, specifically among the cell types where piscidin has shown to be expressed are mast cells, rodlet cells, phagocytic granulocytes and eosinophilic granular cells [43,88,102,104,105]. Interestingly, there is evidence that granulocytes can destroy bacteria in phagosome by intracellular release of piscidin, meaning that piscidin can act against extra and intracellular bacteria [102]. In addition, pleurocidin expression is expressed at 13 days post-hatch in the winter flounder, which is suggested to play an important role in defense during development [101].

Like AMP genes from mammals, piscidin genes can be induced by a variety of stimuli, including Gram-positive and -negative bacteria [78], bacteria cell components like LPS [43,50,58] or the bacterial antigen ASAL [10]. The LPS-mediated induction of epinecidin-1 in zebrafish was shown to require hepatocyte nuclear factor 1 [89]. Furthermore, piscidin genes are induced by parasites [47,104,106], viruses [107], and poly I:C [43,58]. Another study demonstrated that high biomass density (*i.e.*, a higher concentration of fish per volume water in an experimental tank) used as an acute stressor component, led to an to up-regulation of dicentracin in gills and skin as well [74].

Besides microorganisms, piscidin-mediated anti-tumor activity has been shown by the growth inhibition and/or killing of a variety of different cancer-derived cell lines like A549 [108], HT1080 [108,109,110], U937 [111], HL60 [112], U937 [110], HeLa [110] and different breast cancer-derived cell lines including MDA-MB-468, T47-D, SKBR3, MCF7, MCF7-TX400 (paclitaxel-resistant MCF7), MDA-MB-231 and 4T1 [113]. Furthermore, pleurocidin is able to kill breast cancer xenografts in NOD SCID mice, where cell death was caused by mitochondrial membrane damage and ROS production [113]. In addition, disruption of cancer cell membrane has been also shown to occur [110]. Moreover, *in vitro* inhibition of proliferation of U937 and HT1080 was suggested to occur by inducing apoptosis in response to cytokine production like TNF-α, IL-10, IL-15, IL-6, the tumor suppressor p53 [111], and caspases [110]. Also, pleurocidin showed the ability to inhibit HT1080 migration in a dose-dependent manner [109] as well as the rapid killing of a human leukemia cell line [112]. In contrast, pleurocidin showed no lysis of human dermal fibroblasts, umbilical vein endothelial cells and erythrocytes [113].

Several studies have shown that piscidin can disrupt the plasma membranes and cause cellular material efflux by pore formation [114,115]. However, use of membrane models has suggested that membrane composition is an important factor in the lytic capacity of piscidin to disrupt cell membranes [116]. In addition, using site-specific high-resolution solid-state NMR orientational restraints and circular dichroism it was shown that piscidin-1 and -3 induce a membrane-AMP interaction by parallel orientation of the α-helical in membrane model surfaces where fast and large amplitude backbone motions occur [117,118]. Moreover, at very low inhibitory concentrations piscidin does not cause significant cell membrane damage but is capable to inhibit macromolecular synthesis in bacteria [119]. Against fungi, pleurocidin was active against *C. albicans* by causing protoplast regeneration and membrane disruption [95,112] and it has been suggested to cause oxidative stress, triggering apoptosis in *C. albicans* by inducing intracellular reactive oxygen species (ROS) and activation of metacaspases, leading to externalization of phosphatidylserine [94].

Among other attractive features of piscidin includes their ability to retain antibacterial activity at high salt concentrations [41], thermostability (piscidin from seahorse brooding pouch retained full activity after exposing from 20–80 °C for 30 min, and only 20% loss of activity when boiling at 100 °C for 30 min) [75], and relatively low cytotoxicity against mammalian cells [120]. However, in tilapia some piscidin isoforms were hemolytic for tilapia red blood cells. The peptide with the greatest hemolysis activity was also the one with the best antibacterial activity, which is associated with the amphiphilic α-helical cationic structure [78].

The immunomodulatory capacity of piscidins is another feature that has been widely assessed. In fish, they are able to modulate the expression of pro-inflammatory and other immune-related genes like IL-1β, IL-10, IL-22, IL-26, TNF-α, IFN-γ, NF-κB, lysozyme, NOS2, MyD88, TLR4a, TLR1, TLR3, [121,122,123,124,125]. Moreover, in mice this immunomodulatory effect also has been observed, with the modulation of the genes encoding IL-6, IL-10, IL-12, MCP-1, TNF-α, IFN-γ and IgG1 [126,127,128]. Recently, some pleurocidins have shown to be able stimulate human mast cell chemotaxis increasing Ca_2_+ mobilization, and inducing the production of pro-inflammatory cytokines (like CCL2, 1β/CCL4) in mast cells, which was suggested to occur through G-proteins. In addition, it is able to cause mast cells to adhere, migrate, degranulate, and release cysteinyl leukotrienes and prostaglandin D2 [129].

Overall, it appears that piscidins represent an evolutionarily conserved family of peptides, which, while unique to fish, exhibit broad homology to the linear, amphipathic classes of antimicrobial peptides found in many other species.

## 3. β-Defensins

**Figure 2:**
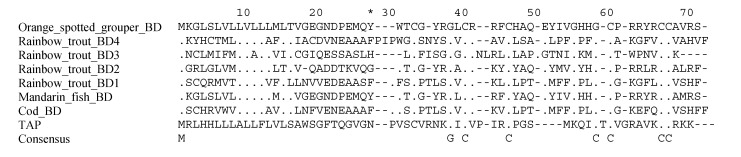
Alignment of β-defensins. Precursor peptide sequences were obtained from published data and from the PubMed protein database, and were aligned using MacVector software. The bovine β-defensin, Tracheal Antimicrobial Peptide (TAP) is shown for comparison. The conserved β-defensin cysteine spacing is shown in the consensus line. The first residue of the mature peptide region (based on the isolated TAP sequence) is denoted with an asterisk.

A general term for cysteine-rich, cationic antimicrobial peptides found in plants, fungi, invertebrates and vertebrates [130,131,132], defensins exhibit a general conformation made by cysteine-stabilized α-helical and β–sheet folds (reviewed in [131,133]). In mammals three types of defensins have been identified based on their structure, α-, β-, and θ-defensins (last one found only in certain nonhuman primates, including the rhesus macacque) [130,134,135]. However in fish, sequence and structural analysis have revealed that fish defensins are solely β-defensin-like proteins [35,44,51,64] including the conserved 6-cyteine motif (Figure 2). To date, up to four genes and five isoforms of defensins have been found in a single species [57,65], apparently as result of gene duplication events that had occurred in vertebrate β-defensins [133]. Fish defensins were first identified in zebrafish, Fugu and tetraodon by a database mining approach [44], but currently defensins have been identified in many other marine and freshwater fish species (see Table 1). Interestingly, a phylogenetic analysis using defensins from human and fish revealed that hBD-4 is the only human defensin that clustered with fish defensins, suggesting possible similar biological properties [35].

The human β-defensin gene has two exons and one intron, fairly typical of most β-defensin genes. Furthermore, mammalian β-defensins are translated as prepeptides, with the mature peptide sequence immediately downstream from the signal sequence [136]. However, in fish three exons and two introns are found [65], encoding a prepropeptide (including signal peptide, propeptide and mature peptide) comprised of 60 to 77 amino acids, and a mature peptide from 38 to 45 amino acids with cationic nature with a *pI* around 8 (except for those in olive flounder, which are around 4, indicating anionic nature [57]). Due its cationic nature they present a net positive charge that can go from +1 to +5. As with all vertebrate β-defensins, there are six conserved cysteines, although in human and birds these are located in a single exon, while in fish they span two exons [57].

Fish β-defensins have proven to be active against both Gram-negative and -positive bacteria (for specific inhibitory values see Supplementary Table S1), although with rather moderate activity. Exceptions to those reports of MICs in the high µM range are *Planococcus citreus* (Gram-positive) [11] and *Aeromonas hydrophila* (Gram-negative) [52], with low MIC values. Other studies using supernatant of lysates HEK293T cells transfected with β-defensins from the Chinese loach or the gilthead seabream showed significant growth inhibition of the Gram-negative *A. hydrophila* and the Gram-positive *B. subtilis* [30,35]. Moreover, β-defensins are also active against fish-specific viruses such as *Singapore grouper iridovirus* (SGIV), *viral nervous necrosis virus* (VNNV), *haemorrhagic septicaemia virus* (VHSV), and the frog-specific *Rana grylio virus* (RGV) [59,60,64]. In addition it has been shown that the α-defensin human defensin-1 (HD-1) is highly active against VHSV, a salmonid rhabdovirus, causing its inactivation and inhibition [137]. However, no assessment has been carried out testing fish-derived defensins against human viruses so far, nor about their potential mechanism of action. Similarly, there are no published studies examining the activity of fish defensins against parasites. A small number of studies demonstrate the activity of human defensins against parasites, showing, for example, that HD-1 is capable to destroy the parasite *Trypanosoma cruzi* by pore formation and induction of nuclear and mitochondrial DNA fragmentation [138], and that hd-5 is able to reduce *Toxoplasma gondii* viability by aggregation [139]. This is an area with great potential both for fish and human biology. As with parasites, there are no studies related to the antifungal activity of fish defensins, in spite of the many studies showing such activity of β-defensins from other species (e.g., those described in [140,141,142].

In addition to their antimicrobial activities, β-defensins have been shown to exhibit multiple immunomodulatory activities (reviewed in [143]). For example, recombinant mBD4 and hBD2 (both β-defensins in mice and human, respectively) have shown to possess chemotactic activity for CCR6-expressing cells (which include monocytes, dendritic cells and T-lymphocytes), which was confirmed using its chemokine ligand CCL20 that competed with these β-defensins [144]. Similar activity has been observed in a fish homologue. β-defensins from the gilthead seabream exhibited chemotactic activity, showing the capacity to attract head-kidney leukocytes [35]. There is evidence of CCR6 mammalian orthologs in zebrafish [145] and rainbow trout [146] that may help address the mechanism. In addition, chemotactic capacity of HNP1 (human α-defensin) towards trout leucocytes has been shown [147]. Furthermore, a β-defensin from Atlantic cod is capable of stimulating antimicrobial activity in phagocytes [11]. Together, the studies suggest that fish β-defensins function similarly to their mammalian counterparts, contributing to the innate host defense in multiple ways.

In mammals, β-defensin expression was initially identified and studied predominantly in skin and mucosal membranes from respiratory, gastrointestinal and genitourinary tracts (reviewed in [148]. More recently, however, numerous β-defensin isoforms have been identified in sperm, with associations to reproduction being demonstrated [149]. While some β-defensins (mostly hBD-1 and its homologue) are constitutively expressed, the expression of most β-defensins can be induced by a variety of factors, including many innate immune mediators and microbe-associated molecular patterns (reviewed in [150]). Furthermore, their expression is observed not only in adult tissues, but during embryonic development as well [151,152]. In fish, constitutive expression seems to start early in the development probably as part of the need of defense in vulnerable stages that rely significantly in the innate immune response [11,57]. However, it is hard to establish specific expression patterns, because this appears to vary between species and isoform [31,44,59,60,65], although in most of the characterized fish β-defensins, skin is one of the tissues with the highest basal expression [31,35,44,65], a widely distributed feature among vertebrate defensins [148,153]. After skin, head-kidney and spleen are the tissues with also high expression, which are the main immune organs in fish [51,52,65]. Nevertheless, in some studies some isoforms of fish β-defensins have shown to possess a widespread constitutive expression [30,52,65]. Furthermore, high expression in eye has been found, suggesting a relevant role in ocular infections [30,52]. In addition, a study in the orange-spotted grouper suggest a relationship of fish β-defensin and reproduction endocrine regulation, finding an isoform that is exclusively expressed in pituitary and testis, where such expression is up-regulated from intersexual gonad to testis in sex reversal, and a deeper analysis proved that the pituitary-specific POU1F1 transcription binding site and the testis-specific SRY site are responsible for this phenomenon [60]. Fish β-defensin genes are induced by a variety of stimuli including cell wall components like LPS [52,59], β-glucans [31] and peptidoglycan [154]. They are stimulated by bacterial challenges from *A. hydrophila* [30], *Y. ruckeri* [65], *V. anguillarum* [11] and *E. tarda* [57]; and by viral challenges, including SGIV [59] or the TLR-3 agonist poly(I:C), to emulate a viral infection [59,65]. In addition, supplemented diets with the diatom *Naviluca* sp. and the lactobacillus *Lactobacillus sakei* have shown to induced β-defensin in gilthead seabream [155]. Thus, β-defensins in fish are true orthologues, exhibiting both structural and functional similarities to mammalian peptides, as well as their patterns of expression. This further supports the hypothesis that β-defensins are an ancient and highly conserved mechanism of host defense in animal species [133].

## 4. Hepcidins

Hepcidins are cysteine-rich peptides with antimicrobial activity that were first discovered in humans [156,157]. Since then, hepcidins have been identified in many other vertebrates including reptiles, amphibians and fish. Although, in birds the existence of a hepcidin needs to be better confirmed [158]. Fish hepcidin was first identified and isolated from the hybrid striped bass [42] and since then hepcidins have been identified in at least 37 fish species. The general structure of human hepcidin is a β-sheet-composed harpin-shaped with four disulfide bridges (formed by eight cysteines) with an unusual vicinal bridge at the hairpin turn [159], which is also the general structure in fish hepcidin [76,79]. However, sequence analysis of fish hepcidins has shown the presence of hepcidins containing 7, 6 or 4 cysteines [9,48].

**Figure 3:**
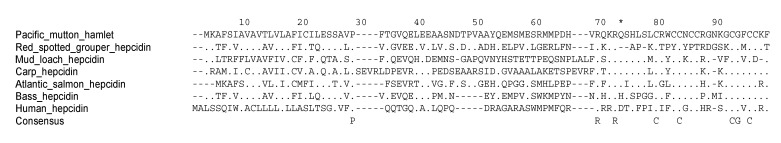
Alignment of hepcidins. Representative precursor peptide sequences were obtained from published data and from the PubMed protein database, and were aligned using MacVector software. Human hepcidin is shown for comparison. The first residue of the mature peptide region is denoted with an asterisk.

Fish hepcidin genes have undergone duplication and diversification processes that have produced multiple gene copies [9], and up to eight copies have been identified [22,34,73]. Hepcidin genes are composed of three exons and two introns encoding a signal peptide, a prodomain and a mature peptide [22,34,72]. The pre-prohepcidin size can range from 81 to 96 amino acids, and the mature hepcidin from 19 to 31, with a molecular weight around 2–3 kDa. Representative sequences are shown in Figure 3. An average *pI* generally above 8 demonstrates their cationic nature. However, a predicted low *pI* of 5.4 of the orange-spotted grouper, indicates the existence of anionic hepcidin [61].

Fish have two types of hepcidins, HAMP1 and HAMP2. However, although HAMP1 is present in actinopterygian and non-actinopterygian fish, HAMP2 has been only found in actinopterygian fish [54,63,158]. Moreover, a phylogenetic study has shown positive Darwinian selection in HAMP2 (but not HAMP1 and its mammalian orthologue) that suggest adaptive evolution probably associated with the host-pathogen interaction in different environments [9,54,160].

Hepcidin expression can be detected as early as in the fertilized egg in blunt snout bream [27] or at 8 h after fertilization in channel catfish [26]. However, in winter flounder and tongue sole it was not detected until day 5 and 6, respectively (larvae stage) [16,37]. Nevertheless, it has been shown that hepcidin isoforms have different expression pattern and kinetics in larval development [70]. In addition, different hepcidin types in a single species can have different rates of expression within the same tissue [80] that can be affected by different stimuli [24,70,73,79]. Interestingly, some isoforms have high basal hepcidin expression in liver, but some have not, where the highest expression often occurs in spleen, kidney and intestine [9,70,73,79].

Similar to other AMP genes, fish hepcidins can be induced by exposure to both Gram-positive and Gram-negative bacteria [12,19,25,26,27,34,36,37,42,48,53,56,61,63,72,73,161,162,163,164,165,166]. Moreover, fungi like *Saccharomyces cerevisiae* [36,61], and tumor cell lines like L-1210 and SAF-1 have shown to induce hepcidin expression as well [36]. Hepcidin genes in fish are also induced by viruses [36,61,73,165], and poly I:C [12,36,164], as well as mitogens [36]. Moreover, environmental estrogenic endocrine disrupting chemicals like 17β-estradiol down-regulates one of the hepcidin isoforms expression in liver in largemouth bass [46].

In humans, hepcidin acts as a type II actue-phase protein [167]. Related to this, time-course experiments under bacterial challenge of fish have shown that the highest expression of hepcidin occurs at 3–6 h and decay after that [23,32,163]. Also, the expression of hepcidin occurs along with other acute phase response proteins like IL-1β, serum amyloid A and precerebellin after infection with *Yersinia ruckeri* in rainbow trout [168]. Related to this, mud loach infected with Gram-negative bacteria showed a high IL-1β-like gene expression-mediated response [56]. Together, these results suggest that hepcidins can also act as a type II acute phase protein, and function as part of a broad innate immune response in fish.

Fish hepcidins are active against a wide variety of bacteria, both Gram-positive and -negative at the low µM range, including potent activity against a large number of fish pathogens (see Supplementary Table S1 for a complete listing). This includes rapid killing kinetics against *S. aureus* and *Pseudomonas stutzeri* [23,62]. Furthermore, synergy between bass hepcidin and moronecidin against *S. iniae* and *Y. enterocolitica* has been demonstrated [161]. In addition, they are active against a number of viruses [80,99,169,170,171], and a recent study indicates that human Hepc25 is able to affect HCV replication in cell culture by inducing STAT3 activation leading to an antiviral state of the cell [172]. In contrast, their quantified activity against fungi appears to be rather low [23,48,161].

A few studies have tried to elucidate the mechanism of action of hepcidin against bacteria. With human Hepc25, the lack of SYTOX uptake showed that membrane permeabilization does not occur [173] in contrast to most antimicrobial peptides [150]. Similar results have been observed with fish peptides, using light emission kinetics, which showed that Medaka recombinant pro-hepcidin and synthetic hepcidin also do not cause membrane permeabilization in *E. coli* [171]. However, human Hepc25 has shown binding to DNA with high efficiency in a retardation assay [173].

Fish hepcidins have also shown the capacity of affect cancer cells viability. For example, tilapia hepcidin TH2-3, have shown inhibition of proliferation and migration of human fibrosarcoma cell line HT1080a in a concentration-dependent manner. Furthermore, TH2-3 was able to cause cell membrane disruption in HT1080 and results also suggest that TH2-3 down-regulates c-Jun leading to apoptosis [174]. TH1-5 inhibit the proliferation of tumor cells (HeLa, HT1080 and HepG2) altering membrane structure and inducing apoptosis at low dose. Also, TH1-5 showed modulation of immune-related genes [175]. A study with medaka hepcidin showed 40% decrease in HepG2 cell viability by addition of 25 and 5 μM of synthetic mature Om-hep1 and recombinant pro-Om-hep1 (prohepcidin), respectively [171]. Interestingly, Pro-Omhep1 has better anti-tumor activity compared with Om-hep1, using HepG2 cells [171].

Fish hepcidins have also shown the ability to modulate the expression of different immune-related genes not only in fish but also in mice. Transgenic zebrafish expressing TH1-5 showed upregulation of IL-10, IL-21, IL-22, lysozyme, TLR-1, TLR-3, TNF-α and NF-κB [176]. However, TH2-3 showed to downregulate some of those upregulated by TH1-5 [177]. In the same context, TH2-3 reduced the amount of TNF-α, IL-1α, IL-1β, IL-6 and COX-2 in mouse macrophages challenged with LPS [178]. Related to this, in turbot it has been shown that hepcidin is able to increase the activation of NF-κB (which control a variety of inflammatory cytokines) through an undetermined yet signaling pathway [165]. TH2-3 have also shown to be able to modulate protein kinase C isoforms in the mouse macrophage RAW264.7 cell line [179], and was also capable to induce morphology changes in these cells similar to PMA-induced changes [179]. Moreover, TH1-5 modulates the expression of certain interferons and annexin (viral-responsive genes) in pancreatic necrosis virus-infected fish [170].

However, despite the potential antimicrobial and immunomodulatory effect, hepcidin is better known for being a key iron regulator controlling ferroportin, which is able to degrade by its internalization, which decrease iron transfer into blood [180]. In fish, although ferroportin internalization by hepcidin has not been proven yet, there is evidence suggesting that fish hepcidin also controls iron [34,56,73,80,162,165,166,181]. It may also serve as a pleiotropic sensor for other divalent metals, because it is up-regulated by exposure to other metals like copper [56] and cadmium [182], which can be considered waterborne or toxic.

## 5. Cathelicidins

**Figure 4:**
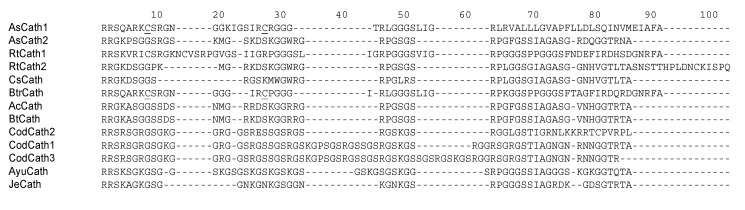
Alignment of fish cathelicidins. Mature peptide sequences were obtained from published data and from the PubMed protein database, and were aligned using MacVector software. Characteristic cysteine residues found in certain classes of fish cathelicidins are underlined. As, Atlantic salmon; Rt, Rainbow trout; Cs, Chinook salmon; Btr, Brown trout; Ac, Arctic char; Bt, Brook trout; Je, Japanese eel.

Unusual among the AMPs, cathelicidins share little sequence homology between the mature peptides. Rather, they are defined by a homologous *N*-terminal region of the precursor peptide, called a cathelin domain, found just after a conserved signal domain (reviewed in [183]). The active, mature peptide is released upon protolytic cleavage by elastase and possibly other enzymes [184]. In mammals, the mature AMP sequence varies greatly, not only between species but also among the often multiple cathelicidin peptides within a single species. In general, however, all mammalian cathelicidin mature peptides are cationic and exhibit an amphipathic characteristic, as well as broad-spectrum antimicrobial activity *in vitro*. As can be seen by the alignment of primary amino acid sequences in Figure 4, there are significant sequence similarities in the *C*-terminal region, and in several short domains, which are highly cationic and glycine-rich.

The first cathelicidins identified in fish were initially isolated as antimicrobial peptides from the Atlantic hagfish, *Myxine glutinosa* [15]. Upon sequence analysis of the cDNA that encoded these peptides, it was discovered that they exhibited homology to cathelicidins, previously only found in mammals to this point. As cathelicidins were discovered in other more conventional fish species, primarily by sequence homology from the cathelin region (e.g., [17,28,77,185]), or more recently by peptide isolation [186], new patterns emerged. In some of the more recently studied fish cathelicidins, while a high degree of homology is maintained in the cathelin domain (see [185] for a comprehensive alignment), there appears to be a higher degree of sequence similarity of the mature peptide than seen in mammals. Thus, fish cathelicidins can now be subdivided into two classes—the linear peptides, and those that exhibit a characteristic disulphide bond. In contrast to mammalian cathelicidins, there is significant sequence homology among members of the classes (up to 90%), and little homology between the classes. In addition, the recently identified cathelicidins found in cod appear to comprise a third class, based on sequence homology between themselves, and a lack of homology with either of the other two classes [185]. An alignment of representative fish cathelicidins is shown in Figure 4.

As cathelicidin peptides are purified from more species, their *in vitro* antibacterial activities appear to exhibit significant variability with respect to selectivity, depending on the species. For example, cod cathelicidin (codCATH) is highly active against those Gram-negative bacterial species examined, but almost inactive against the Gram-positive species. It also exhibits potent antifungal activity against *C. albicans* [13]. In contrast, the hagfish cathelicidins are active against both Gram-positive and -negative bacteria, but inactive against Candida [15]. Even more specifically, rainbow trout cathelicidins are active against *Y. ruckeri*, while Atlantic salmon cathelicidins are not [187]. Thus, the variability in the mature peptide sequence of these molecules appears to direct the antimicrobial activities, and is probably a result of an evolutionary divergence to address specific pathogens.

Based on their antimicrobial activity, most of the work to elucidate their role *in vivo* has examined the expression of the cathelicidin genes in the various fish species with respect to induction by innate immune regulators, such as bacteria and to different pathogen-associated molecular patterns (PAMPs). Importantly, cathelicidin expression is observed early in embryonic development, suggesting that its role in immunity is present early on [186]. *In vitro*, both bacteria and bacterial DNA were sufficient to induce cathelicidin expression in a cultured embryonic salmon cell line [188], suggesting that like mammalian cathelicidins, the fish homologs play a similar role in antibacterial host defense. Surprisingly, purified LPS (that is, treated with DNase I) could not induce the gene. This regulation has been further elucidated by the demonstration of a wide variability of the Chinook salmon embryonic cell line’s response to different bacteria and to poly I:C, LPS and flagellin [189]. Further *in vitro* evidence of this role was demonstrated by the induction of rainbow trout cathelicidin in a macrophage cell line from that species by IL-6, an important mediator of the innate immune response [190], and by a novel TNF-α isoform [191]. Similarly, stimulation of a cell line from Atlantic cod with poly I:C induced expression of the gmCath1 (from cod) gene promoter [192]. In addition to bacteria and their products, trout cell lines were shown to induce cathelicidin gene expression upon incubation with the oomycete *Saprolegnia parasitica* [193].

*In vivo* studies further support this hypothesis. When ayu were injected with live pathogenic bacteria, there was a time-dependent induction of cathelicidin expression in numerous tissues, including gill, liver, spleen and intestine [20]. Further, Atlantic salmon and rainbow trout infected with *Y. ruckeri* led to an induction in cathelicidin expression [187,194]. In the Atlantic cod, a difference in inducibility was observed. Cathelicidin experession in the gills was induced by a 3-h incubation with *Aeromonas salmonicida*, but not *V. anguillarum*, suggesting a more complex role is played by these peptides in host defense.

In mammals, cathelicidins have been demonstrated to exhibit multiple activities, both immune and non-immune, well in excess of their *in vitro* antimicrobial activities (reviewed in [195]). While research in fish has not approached this level, a recent study demonstrated that two Atlantic salmon cathelicidins induced the rapid and transient expression of IL-8 in peripheral blood leukocytes [187]. This suggests that the immunomodulatory activities seen by mammalian cathelicidins may be shared by their fish counterparts, and may thus be an evolutionarily conserved mechanism of innate immune regulation.

## 6. Histone-Derived Peptides

**Figure 5:**
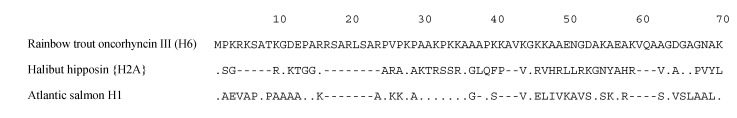
Alignment of histone-derived peptides. Representative peptide sequences were obtained from published data and from the PubMed protein database, and were aligned using MacVector software. Since the sequences are homolgous to different histone peptide fragments, there is no shared sequence homology.

While examining an amphibian species for novel antimicrobial peptides, Park *et al*. [196] described a new peptide from the Asian toad, *Bufo bufo gargarizans*, which they called Buforin I. This turned out to be identical to the *N*-terminal portion of histone 2A. This led to the demonstration of antimicrobial activity of histone fragments from numerous species (reviewed in [197]), suggesting that these proteolytic fragments are part of an ancient innate immune mechanism. Histone-derived AMPs have been identified in a number of fish species, with broad-spectrum activity against both human and fish pathogens (reviewed in [198]), including water molds [199] and a parasitic dinoflagellate [69]. They are expressed and secreted in fish skin, and found in other tissues, including gill, speen and the gut. They are not limited to the *N*-terminus of the histones, as was found for the Buforin peptide, but can be found as fragments of both termini, from histones H1, H2A, H2B and H6 (see Figure 5). Further evidence that they play a role in host defense of the fish comes from studies showing that expression of histone-derived AMP genes are induced under conditions of stress in specific tissues of different fish species [74,200].

## 7. Therapeutics

All AMPs have common characteristics that support their development as therapeutic antimicrobials. These include broad-spectrum activity against a wide variety of pathogens; potent activity under a wide range of conditions, including temperature and in secretions such as saliva; and a reduced capacity to the development of resistance by bacteria. The identification and characterization of peptides from fish has provided a unique contribution in this arena. While the structural characteristics of fish peptides do not appear particularly different from their mammalian, insect or amphibian homologues, there may be specific differences with respect to activity. It appears that overall their antimicrobial activities against human pathogens is in the same range as AMPs from other species. However, it is possible that they are more active against fish pathogens, as they most likely have evolved together with those pathogens. It is difficult to know this, however, as few studies have compared non-fish peptides with fish peptides against fish pathogens. One area where fish peptides may provide an advantage is in food preservation [201], as they are derived from a natural food source, and thus may be more amenable to being consumed.

Since many AMPs are sensitive to high salt concentrations [202,203,204], the ability of some fish AMPs to kill microbes even at extremely high salt concentrations, such as those found in the marine environment, make them important targets for investigation. Pleurocidin, for example, maintains its antibacterial activity even up to 300 mM NaCl [6], similar to other piscidins [41,205]. Understanding the structural foundation that supports this salt-independent activity could aid in the design of novel peptides [206] or mimetics that could address infections under a wide range of normal and abnormal salt concentrations, whether in serum, tear film hyperosmolarity, or in saliva to address dental caries [207]. In addition to their potential uses as antimicrobials, some fish AMPs have been observed to exhibit *in vitro* cytotoxic activity against a variety of cancer cells [113,208].

Different applications of piscidin have been promising. For example, epinecidin-1, when administrated orally or injected (in pre-, co- and post-infection) can significantly enhance survival in zebrafish and grouper that were challenged with *Vibrio vulnificus* [58,123,124]. Related to this, electrotransfer of epinecidin-1 in zebrafish and grouper muscle showed significant reduction in *V. vulnificus* and *Streptococcus agalactiae* bacterial counts [121,122,125]. Moreover, treatment of lethally-challenged methicillin-resistant *S. aureus* (MRSA) mice with epinecidin-1 allowed mice to survive by decreasing considerably the bacterial counts, where also there was evidence of wound closure and angiogenesis enhancement [128].

In oral disease treatment piscidins are also promising due to the potent effect of chrysophsin-1 in killing the cariogenic pathogen *Streptococcus mutans* [209]. Furthermore, pleurocidin also demonstrated anti-cariogenic activity by being able to kill both *S. mutans* and *S. sobrinus*, where killing of biofilms occurred in a dose-dependent manner. In addition, it showed to retained its activity in physiological or higher salt concentration, and was relatively stable in presence of human saliva and no hemolysis was found [207,210].

Epinecidin-1 showed to be a potential candidate for topical application that can prevent vaginal or skin infections due to the synergistic effect that possess with commercial cleaning solutions, where such effect was not affected by low pH or after being stored at room temperature and at 4 °C for up to 14 days [211]. The synergistic effect of pleurocidin and several antibiotics [212], bacteroricin [213] and histone-derived [214] has also been shown [212,213]. Furthermore, the creation of antimicrobial surfaces has been made by the immobilization of chrysophsin-1 resulting in a surface with antibacterial activity capable to killed around 82% of *E. coli* bacteria [215].

A recent interest finding is the ability of epinecidin-1 to create inactivated virus for vaccination purposes. Huang *et al*. found that mice injected with Epi-1-based inactivated Japanese encephalitis virus (JEV) reached 100% survival (in a dose-dependent manner), and the performance was better than the formalin-based JEV-inactivated vaccine. This was caused by the modulation of immune-related genes, including the increase of anti-JEV-neutralizing antibodies in serum, which suppressed the multiplication of JEV in brain sections [126].

Fish hepcidins are also under examination for development as therapeutics. One example of this is the study carried out by Pan *et al*. [216], where injections with pre-incubated tilapia hepcidin TH2-3 and 10^8^ cfu of *Vibrio vulnificus* for 30 min enhanced the survival of infected and re-infected mice, obtaining up to 60% of survival with a dose of 40 μg/mice. In addition, TH2-3 also showed significant prophylactic effect by administration prior infection, where survival rates of 100% were obtained after 7 days of infection. Also, curative effects were shown when fish were first infected and later injected with 40 μg/mice of TH2-3, obtaining survival rates up to 50%. But more interesting, was the fact that TH2-3 had better bacteriostatic effect than tetracycline in controlling the bacterial burden in blood, although in liver there was no significant difference, demonstrating the capacity of TH2-3 to control multiplication of *V. vulnificus* in mice. Although the direct *in vivo* TH2-3-mediated killing was not confirmed, a microarray analysis showed that TH2-3 clearly altered the gene expression profiles improving the host response in mice [216]. In addition, a transgenic zebrafish expressing TH2-3 showed to be able to decrease *V. vulnificus* burden significantly, but not *S. agalactiae* [177]. Zebrafish expressing TH1-5 exhibited enhanced bacterial resistance by decreasing the bacterial burden of both same pathogens [176]. In addition, TH1-5 has showed to be effective at increasing survival and decreasing the number of infectious bacteria in ducks challenged with *Riemerella anatipestifer*, which also showed to be able to modulate the expression of immune-related genes [114].

However, as with other AMPs, they share the similar problems that hinder their further development, especially for use in human medicine. These include a tendency to be inactivated in the body, increased expense of peptide synthesis, and sensitivity to protease digestion. Attempts to address these issues with fish peptides, include the identification of smaller peptide fragments that might exhibit better activity with smaller molecules [217], and the observation of high levels of synergy with bacterial AMPs [213,214], as well as conventional antibiotics [218] allowing for reduced concentrations. One strategy that may have some success is the design of small molecule peptide mimetics that incorporate the structural characteristics of the peptides necessary for their activity (reviewed in [219]). Initial *in vitro* and *in vivo* results with molecules designed from magainins and defensins have been encouraging, demonstrating antibacterial [220] and antifungal [221] activities, as well as immunomodulatory activity [222]. Another strategy that has been examined extensively in other species (reviewed in [223]) is the use of exogenous agents to modulate the expression of endogenous AMPs in the fish. Terova *et al*. have demonstrated the induction of an AMP initially isolated from sea bass [33] by feeding them a cell wall extract from *S. cerevisiae* [224], suggesting a novel method for enhancing the natural defense mechanism of the fish. Incorporation of an enhancer of AMP expression in the diet could be a cost-effective part of an overall strategy of modulating the innate immune system of the fish to control infection in aquaculture (reviewed in [198]).

## 8. Conclusions

The comprehensive characterization of AMPs from fish, on the structural, genetic and functional levels, has provided a wealth of information. Examination of AMPs in a single species, such as the Atlantic cod, where members of all five groups of AMPs have been identified can help understand the role of these peptides in innate host defense of the fish. Studies on the similarities and differences with peptides from non-fish species contribute to our understanding of the evolutionary relationships of innate host defense mechanisms among vertebrates. Furthermore, they can provide important information for the better design of novel therapeutic agents, both for microbial infections as well as cancer and other conditions. Unique for the field of fish AMPs is the potential application to aquaculture. The constant risk of large-scale microbial infection that can lead to significant economic losses demands new strategies to prevent or treat these pathogens. Many of the studies described above have demonstrated that specific fish AMPs have potent activity against fish pathogens. Furthermore, other studies have shown that some pathogens induce potent innate immune responses in the fish. Complicating this is the evolutionary battle with the pathogens. For example, challenge of Atlantic cod with the pathogen *V. anguillarum* induces the expression of a β-defensin, which is antibacterial against other species, but not the inducing *V. anguillarum* [11]. Thus, the large body of work described above provides a solid foundation for strong future work to better understand both the role of these peptides in host defense of the fish, as well as the development of these peptides and their derivatives as potential therapeutics.

## Supplementary Files

**Table S1:** Antimicrobial activity of fish AMPs. Bacteria and fungi included MICs (μM, or μg/mL will be indicated) values, MLCs (μM), vLD_90_ (virtual 90% lethal dose, mg/mL [mean ± SEM]) or *IC*_50_ (μM). If values are not included antimicrobial activity was assessed by inhibitory halo (IH) and size in millimeter was not presented, if so, is indicated. If isoforms were tested, the best performance value was used. For parasites, the minimum protozoacidal concentration (PC_min_) or MIC is indicated. Habitat of microorganisms are indicated (freshwater, marine or other).

Bacteria	β-Defensin	Piscidin	Hepcdin	Cathelicidin	Histone-derived	Pathogenic		Habitat
						Fish	Human	
Gram negative								
*Escherichia coli*	39.0 ± 3.7 (vLD_90_) [60] 32.6 ± 1.5 (vLD_90_) [52] IH [10]	5–10 μM (MIC) [50] 2–10 μM (MIC) [117] 50 μg/mL (MIC) [40] 2.2–3.3 μM (MIC) [6] 5 μM (MIC) [43] 3–6 μM (MIC) [47] 2 μg/mL (MIC) [83] 3 μM (MIC) [75] 1 μg/mL (MIC) [8]	5–10 μM (MIC) [23] >96 μM (MIC) [62] 6-12 μM (MIC) [171] 18.66 (IC_50_) [36] 11 μM (MIC) [161] 12–24 μM (MIC) [48] >8.18 μM (MIC) [37] 34.56 mm^2^ (IH) [49]	<1 μg/mL (MIC) [187] 1 μg/mL (MIC) [225] [20] 2–4 μg/mL (MIC) [77] 5 μg/mL (MIC) [186]	1 μg/mL(MIC) [29] >10 μg/mL(MIC) [14] 2.5 μg/mL (MIC) [38]	X	X	Freshwater and others
*Plesiomonas shigelloides*			11 μM (MIC) [161]			X	X	Freshwater
*Klebsiella pneumoniae*		12.6 μg/mL (MIC) [82] 2.5–5 μM (MIC) [41]	22 μM (MIC) [161]	18.8 μM (MIC) [77]			X	Other
*Klebsiella oxytoca*		5–10 μM (MIC) [41] 12.5 μg/mL (MIC) [58]	>44 μM (MIC) [161]				X	Other
*Shigella sonnei*		5–10 μM (MIC) [41]	>44 μM (MIC) [161]				X	Other
*Shigella flexneri*		3.1 μg/mL (MIC) [40] 2.5–5 μM (MIC) [41]	>96 μM (MIC) [62] 22 μM (MIC) [161]				X	Freshwater and others
*Yersinia enterocolitica*		2.5–5 μM (MIC) [41] 100 μM (MIC) [58]	22 μM (MIC) [161]			X	X	Freshwater and others
*Yersinia ruckeri*		20–40 μM (MIC) [50]				X		Freshwater
*Aeromonas salmonicida*	>50 μM (MIC) [11]	17.7–35 μM (MIC) [6] 2 μg/mL (MIC) [83] 1 μg/mL (MIC) [8] 5 μM (MLC) [71]	>44 μM (MIC) [161]	9.4 μg/mL (MIC)[77] 1–5 μg/mL (MIC) [225] 50 μg/mL (MIC) [20] 10 μg/mL (MIC) [186]	20 μg/mL (MIC) [38] >1.2 μg/mL (MIC) [66]	X		Freshwater and marine
*Aeromonas hydrophila*	13.4 ± 0.7 [52] IH [51]	1.05 μg/mL (MIC) [78] 19.78 μg/mL (MIC) [78] >21.4 μg/mL (MIC) [78] >160 [50] >20 [41] >96 [47]	10–20 μM (MIC) [23] >96 μM (MIC) [62] 1.5–3 μM (MIC) [171] >44 μM (MIC) [161] 3–6 μM (MIC) [48] 31.66 mm^2^ (IH) [49]			X	X	Freshwater and others
*Aeromonas sobria*	59.4 ± 8.8 [60]	10–20 μM (MIC) [50]			2.5 μM (MIC) [38]	X	X	Freshwater and others
*Aeromonas punctata*		10–20 μM (MIC) [50]				X		Freshwater and others
*Vibrio anguillarum*	>50 μM (MIC) [11] 58.2 ± 23.4 (vLD_90_) [60] 21.1 ± 1 (vLD_90_) [52]	20–40 μM (MIC) [50] 6.3 μg/mL (MIC) [40] 1.25 μM (MLC) [71]	2.92 μM (MIC) [37]	5 μM (MIC) [187] 0.5–2.5 μM (MIC) [225] 5 μM (MIC) [186]		X		Marine
*Vibrio parahaemolyticus*			>60 μM (MIC) [23] >96 μM (MIC) [62] >48 μM (MIC) [171] 3–6 μM (MIC) [48] 5.84 μM (MIC) [37] 94.25 mm^2^ (IH) [49]	25 [20]		X	X	Marine
*Vibrio fluvialis*	43.0 ± 10 (vLD_90_) [60] 35.1 ± 2.7 (vLD_90_) [52]		>96 μM (MIC) [62] 123.11 mm^2^ (IH) [49]	3.1 μg/mL (MIC) [20]		X	X	Marine
*Vibrio harveyi*		12.5 μg/mL (MIC) [58] 5 μM (MLC) [71]	20–40 μM (MIC) [23] >96 μM (MIC) [62] 6–12 μM (MIC) [48] 5.84 μM (MIC) [37] 113.10 mm^2^ (IH) [49]	6.2 μg/mL (MIC) [20]		X		Marine
*Vibrio alginolyticus*		0.03 μg/mL (MIC) [78] 1.24 μg/mL (MIC) [78] 2.68 μg/mL (MIC) [78]	>60 μM (MIC) [23] >96 μM (MIC) [62] >48 μM (MIC) [171] 12–24 μM (MIC) [48] 118.06 mm^2^ (IH) [49]			X	X	Marine
*Vibrio vulnificus*		0.03 μg/mL (MIC) [78] 0.62 μg/mL (MIC) [78] 0.67 μg/mL (MIC) [78] 6.25 μg/mL (MIC) [58] 2.5 μM (MLC) [71]	20 μM (MIC) [61,163]			X	X	Marine
*Vibrio cholera*		2.5–5 μM (MIC) [41]				X	X	Freshwater, marine and others
*Vibrio damsela*		48 μM (MIC) [75]				X	X	Marine
*Vibrio penaeicida*		5 μM (MLC) [71]						Marine
*Salinivibrio costicola*		3.125 μg/mL (MIC) [58]						Marine and others
*Edwardsiella tarda*			>44 μM (MIC) [161] 2.92 μM (MIC) [37]			X	X	Marine and freshwater
*Riemerella anatipestifer*		6.25 μg/mL (MIC) [114]	25 μg/mL (MIC) [114]					Other
*Pseudomonas aeruginosa*	44.5 ± 11.8 [60]	0.52 μg/mL (MIC) [78] >19.78 μg/mL (MIC) [78] 10.70 μg/mL (MIC) [78] 60 μg/mL (MIC) [58] 1 μg/mL (MIC) [83] 28 μg/mL (MIC) [82] >35 μM (MIC) [6] 5–10 μM (MIC) [41]	>44 μM (MIC) [161] >96 μM (MIC) [62]	12.5 μg/mL (MIC) [20] 1–4 μg/mL (MIC) [77]		X	X	Freshwater, marine and others
*Pseudomonas fluorescens*		10–20 μM (MIC) [50] 1.5–3 μM (MIC) [47]	24–48 μM (MIC) [62]		20 μM (MIC) [38]	X	X	Freshwater and others
*Pseudomonas stutzeri*			<1.5 μM (MIC) [62] 3–6 μM (MIC) [171]				X	Other
*Enterobacter cloacae*		10–20 μM (MIC) [41] 100 μg/mL (MIC) [58]	>44 μM (MIC) [161]			X	X	Freshwater and others
*Enterobacter aerogenes*		10–20 μM (MIC) [41] 25 μg/mL (MIC) [58]					X	Others
*Salmonella arizonae*		10–20 μM (MIC) [41]	>44 μM (MIC) [161]				X	Other
*Salmonella choleraesuis*		10–20 μM (MIC) [41] 6 μM (MIC) [75]	>44 μM (MIC) [161]				X	Other
*Salmonella typhimurium*		8.8–17.7 μM (MIC) [6] 10–20 μM (MIC) [41] 2 μg/mL (MIC) [83] 1 μg/mL (MIC) [8]	>44 μM (MIC) [161]		2 μM (MIC) [29]		X	Other
*Serratia marcescens*		>35 μM (MIC) [6] >20 μM (MIC) [41]	>44 μM (MIC) [161]		4 μM (MIC) [29]		X	Other
*Cytophaga columnare*		40–80 μM (MIC) [50]				X		Freshwater
*Cytophaga aquatilis*		2.2–4.4 μM (MIC) [6]				X		Freshwater
*Proteus vulgaris*		2–10 μM (MIC) [117]X				X	X	Freshwater and others
*Photobacterium damsela subsp. piscidida*		1.5 μg/mL (MIC) [40]				X		Marine and freshwater
*Pasteurella haemolytica*		4.4–8.8 μM (MIC) [6]					X	Other
*Burkholderia cepacia*		>20 μM (MIC) [41]					X	Freshwater and others
*Moraxella catarrhalis*		2.5–5 μM (MIC) [41]					X	Other
Neisseria gonorrhoeae		>20 μM (MIC) [41]					X	Other
*Psychrobacter* sp.		10 μM (MIC) [43]						Marine and others
*Morganella morganii*		12 μM (MIC) [75]					X	Other
*Enterococcus faecium*		15 μM (MIC) [75]					X	Other
**Gram positive**								
*Micrococcus luteus*	25–50 μM (MIC) [11] 296.5 ± 65.5 (vLD_90_) [60] 311 ± 15.6 (vLD_90_) [52]	10–20 μM (MIC) [41] 3.125 μg/mL (MIC) [58]	>96 μM (MIC) [62] 1.5–3 μM (MIC) [48] 2.5–5 μM (MIC) [23] 24–48 μM (MIC) [62] 34.56 mm^2^ (IH) [49]				X	Freshwater and others
*Staphylococcus aureus*	358.5 ± 46.5 (vLD_90_) [60] 229.8 ± 12.8 (vLD_90_) [52] IH [51]	0–2 μM (MIC) [117] 3.1 μg/mL (MIC) [40] 17.7–35 μM (MIC) [6] 62.5 μg/mL (MIC) [82] 1.25–2.5 μM (MIC) [41] 6–12 μM (MIC) [47] 6.25 μg/mL (MIC) [58] 8 μg/mL (MIC) [83] 1.5 μM (MIC) [75]	1.25–2.5 μM (MIC) [53] 1.5–3 μM (MIC) [62] 1.5–3 μM (MIC) [171] >44 μM (MIC) [161] 3–6 μM (MIC) [48] 1.25–2.5 μM (MIC) [23] >8.18 μM (MIC) [37] 47.12 mm^2^ (IH) [49]	11–45 μM (MIC) [77]	2μM (MIC) [29]		X	Other
*Staphylococcus epidermidis*		5–10 μM (MIC) [41] 12.5 μg/mL (MIC) [58] 8 μg/mL (MIC) [83] 4 μg/mL (MIC) [8]	20–40 μM (MIC) [23] >96 μM (MIC) [62] 6–12 μM (MIC) [48]		10 μM (MIC) [38]		X	Other
*Staphylococcus saprophiticus*		5–10 μM (MIC) [41] 7.5 μM (MIC) [75]					X	Other
*Staphylococcus haemolyticus*		7.5 μM (MIC) [75]					X	Other
*Staphylococcus xylosus*		>20 μM (MIC) [41] 50 μg/mL (MIC) [58]						Other
*Staphylococcus warneri*		15 μM (MIC) [75]						Other
*Bacillus subtilis*		1.1–2.2 μM (MIC) [6] 0.75–1.5 μM (MIC) [47] 48 μM (MIC) [75]	5–10 μM (MIC) [23] >96 μM (MIC) [62] 11.41 (IC_50_) [36] 3–6 μM (MIC) [48] 28.86 mm^2^ (IH) [49]		11 μg/mL (MIC) [29] 0.6 μg/mL (MIC) [66] 1.3 μg/mL (MIC) [38]			Other
*Bacillus cereus*	17.5 ± 4 (vLD_90_) [60]	0–2 μM (MIC) [117] 5 μM (MIC) [43]	40–60 μM (MIC) [23] >96 μM (MIC) [62] 12–24 μM (MIC) [48]				X	Other
*Corynebacterium glutamicum*			2.5–5 μM (MIC) [23] 48–96 μM (MIC) [62] 3–6 μM (MIC) [171] 1.5–3 μM (MIC) [48]					Other
*Planococcus citreus*	0.4–0.8 μM (MIC) [11]				0.08 μg/mL (MIC) [66]			Marine
*Enterococcus faecalis*		8.39 μg/mL (MIC) [78]	>44 μM (MIC) [161]				X	Other
		9.89 μg/mL (MIC) [78]						
		>21.41 μg/mL (MIC) [78]						
		3.1 μg/mL (MIC) [40]						
		>256 μM (MIC) [210]						
		2.5–5 μM (MIC) [41]						
		8-16 μg/mL (MIC) [209]						
*Listeria monocytogenes*		2.5–5 μM (MIC) [41] 25 μg/mL (MIC) [58]					X	Other
*Streptococcus agalactiae*		0.13 μg/mL (MIC) [78]				X	X	Freshwater, marine and others
		0.31 μg/mL (MIC) [78]						
		0.33 μg/mL (MIC) [78]						
		1.25–2.5 μM (MIC) [41]						
		12.5 μg/mL (MIC) [58]						
*Streptococcus iniae*		3.1 μg/mL (MIC) [40] 1.25–2.5 μM (MIC) [41] 1.5 μM (MLC) [71]				X	X	Freshwater, marine and others
*Streptococcus mutans*		2.2 μM (MIC) [207] 8 μg/mL (MIC) [210] 4 μg/mL (MIC) [209]			1 μg/mL (MIC) [29]		X	Other
*Streptococcus sobrinus*		8 μg/mL (MIC) [210] 4 μg/mL (MIC) [209]					X	Other
*Streptococcus sanguinis*		32 μg/mL (MIC) [210] 4–8 μg/mL (MIC) [209]					X	Other
*Streptococcus gordonii*		8 μg/mL (MIC) [210] 8 μg/mL (MIC) [209]					X	Other
*Streptococcus bovis*		1.25–2.5 μM (MIC) [41]					X	Other
*Streptococcus equisimilis*		2.5–5 μM (MIC) [41]					X	Other
*Streptococcus mitis*		1.25–2.5 μM (MIC) [41]					X	Other
*Streptococcus pneumoniae*		1.25–2.5 μM (MIC) [41] 12.5 μg/mL (MIC) [58]					X	Other
*Streptococcus pyogenes*		1.25–2.5 μM (MIC) [41] 25 μg/mL (MIC) [58]					X	Other
*Lactococcus garviae*		3.1 μg/mL (MIC) [40] 5 μM (MLC) [71]				X	X	Marine and others
*Leucothrix mucor*		>35 μM (MIC) [6]				X		Marine
*Lactobacillus acidophilus*		128 μg/mL (MIC) [210] 4–8 μg/mL (MIC) [209]						Other
*Lactobacillus casei*		32 μg/mL (MIC) [210] 4 μg/mL (MIC) [209]						Other
*Lactobacillus fermenti*		2 μg/mL (MIC) [210] 4 μg/mL (MIC) [209]						Other
*Actinomyces viscosus*		8 μg/mL (MIC) [209]					X	Other
*Actinomyces naeslundii*		8 μg/mL (MIC) [209]					X	Other
**Fungi**								
*Aspergillus fumigatus*		50–100 μM (MIC) [41]					X	Other
*Aspergillus niger*		48–96 μM (MIC) [47]	20–40 μM (MIC) [23] 44 μM (MIC) [161] 12–24 μM (MIC) [48]				X	Other
*Fusarium graminearum*			20–40 μM (MIC) [23] 12–24 μM (MIC) [48]					Other
*Fusarium solani*			20–40 μM (MIC) [23] 12–24 μM (MIC) [48]				X	Other
*Fusarium oxysporum*		0.78–1.56 μM (MIC) [41]				X	X	Freshwater, marine and others
*Fusarium culmorum*		0.39–0.78 [41]						Other
*Candida albicans*		15.4 μg/mL (MIC) [82] 10–20 μM (MIC) [41] 24–48 μM (MIC) [47] 8 μg/mL (MIC) [83] 96 μM (MIC) [75] 4 μg/mL (MIC) [8] 5 μM (MIC) [115] 6.25 μM (MIC) [226]	>60 μM (MIC) [23] >96 μM (MIC) [62] >44 μM (MIC) [161] >48 μM (MIC) [48]	2.3 [77] 2.5 [186]			X	Other
*Candida glabrata*		10–20 μM (MIC) [41]					X	Other
*Candida lusitania*		10–20 μM (MIC) [41]						
*Candida tropicalis*		10–20 μM (MIC) [41]					X	Other
Saccharomyces cerevisiae		384 μM (MIC) [75] 5 μM (MIC) [115]					X	Other
*Pichia pastoris*			>60 μM (MIC) [23] >96 μM (MIC) [62] >48 μM (MIC) [48]					Other
*Saprolegnia* sp		12.5–25 μg/mL (MOC) [98]				X		Freshwater and others
Neurospora crassa		1.56–3.12 μM (MIC) [41]						Other
Trichosporon beigelii		2.5 μM (MIC) [115] 1.56 μM (MIC) [226]					X	Other
*Malassezia furfur*		6.25 μM (MIC) [226]					X	Other
**Parasites**								
*Trichomonas vaginalis*		12.5 μg/mL (MIC) [115]					X	Other
Trichodina		12.5–100 μg/mL (PC_min_) [97]				X		Freshwater and marine
*Cryptocaryon* theront		12.5 μg/mL (PC_min_) [97]				X		Marine
*Amyloodinium* dinospore		6.3 μg/mL (PC_min_) [97]				X		Marine
*Ichthyophthirius* theront		6.3 μg/mL (PC_min_) [97]				X		Freshwater
**Virus**								
VHSV	[64]		[80]			X		Marine
NNV	[59]	[227]	[99,169]			X		Marine
IPNV			[170]			X		Marine
RGV	[60]					X		Marine and Freshwater
SGIV	[59]		[61]			X		Marine
CCV		[100]				X		Freshwater
FV3		[100]						Other
